# Synthesis, In Vitro, and Computational Studies of PTP1B Phosphatase Inhibitors Based on Oxovanadium(IV) and Dioxovanadium(V) Complexes

**DOI:** 10.3390/ijms23137034

**Published:** 2022-06-24

**Authors:** Tomasz Kostrzewa, Jakub Jończyk, Joanna Drzeżdżon, Dagmara Jacewicz, Magdalena Górska-Ponikowska, Marcin Kołaczkowski, Alicja Kuban-Jankowska

**Affiliations:** 1 Department of Medical Chemistry, Faculty of Medicine, Medical University of Gdansk, 80-211 Gdansk, Poland; magdalena.gorska-ponikowska@gumed.edu.pl; 2 Department of Medicinal Chemistry, Faculty of Pharmacy, Jagiellonian University Medical College, 30-688 Krakow, Poland; jakub.jonczyk@uj.edu.pl (J.J.); marcin.kolaczkowski@uj.edu.pl (M.K.); 3 Department of Environmental Technology, Faculty of Chemistry, University of Gdansk, 80-308 Gdansk, Poland; joanna.drzezdzon@ug.edu.pl (J.D.); dagmara.jacewicz@ug.edu.pl (D.J.); 4 IEMEST Istituto Euro-Mediterraneo di Scienza e Tecnologia, 90127 Palermo, Italy; 5 Department of Biophysics, Institute of Biomaterials and Biomolecular Systems, University of Stuttgart, 70174 Stuttgart, Germany

**Keywords:** protein tyrosine phosphatase PTP1B, triple negative breast cancer, oxovanadium(IV) complexes, dioxovanadium(V) complexes

## Abstract

One of the main goals of recent bioinorganic chemistry studies has been to design and synthesize novel substances to treat human diseases. The promising compounds are metal-based and metal ion binding components such as vanadium-based compounds. The potential anticancer action of vanadium-based compounds is one of area of investigation in this field. In this study, we present five oxovanadium(IV) and dioxovanadium(V) complexes as potential PTP1B inhibitors with anticancer activity against the MCF-7 breast cancer cell line, the triple negative MDA-MB-231 breast cancer cell line, and the human keratinocyte HaCaT cell line. We observed that all tested compounds were effective inhibitors of PTP1B, which correlates with anticancer activity. [VO(dipic)(dmbipy)]·2 H_2_O (Compound **4**) and [VOO(dipic)](2-phepyH)·H_2_O (Compound **5**) possessed the greatest inhibitory effect, with IC_50_ 185.4 ± 9.8 and 167.2 ± 8.0 nM, respectively. To obtain a better understanding of the relationship between the structure of the examined compounds and their activity, we performed a computer simulation of their binding inside the active site of PTP1B. We observed a stronger binding of complexes containing dipicolinic acid with PTP1B. Based on our simulations, we suggested that the studied complexes exert their activity by stabilizing the WPD-loop in an open position and limiting access to the P-loop.

## 1. Introduction

Protein phosphorylation plays an important role in cellular processes, including differentiation, proliferation, cell growth, and apoptosis. During mitosis, the structure and organization of cells change rapidly [1]. Consequently, the cytoplasm and organelles are distributed evenly, which results in even genetic segregation and the formation of two viable cells. Protein kinases and phosphatases promote the dynamic and reversible phosphorylation of proteins and constitute an essential regulatory mechanism of cell signaling [2]. Abnormality in cell division can cause aberrant chromosomal cell proliferation, which is a hallmark of tumor and the cause of many congenital defects. Protein tyrosine phosphatase PTP1B is a protein which is involved in the complex enzyme reactions required to dephosphorylate proteins with tyrosine residues. Uncontrolled changes in these processes can lead to malignancies, autoimmune diseases, and metabolic development disorders [3,4,5,6,7,8].

Breast cancer is the most common neoplastic disease among women, with over 2 million affected worldwide [9]. According to the World Health Organization (WHO) and the Union for International Cancer Control (UICC), cancers will soon rank first among the causes of death. Therefore, it is important to look for novel more effective treatment methods to increase the survival rate and life expectancy of patients. On the other hand, searching and synthesizing of new compounds with selective inhibitory properties against pro-oncogenic proteins with correlation to cytotoxic activity is both a challenge and an important path in research on new potential drugs. One of the molecular targets in the research on selective anti-cancer compounds may be protein tyrosine phosphatases.

Protein tyrosine phosphatases (PTPs) play an essential role in many signal transduction pathways [2,10], and the identification of PTPs inhibitors can be a potential therapeutic target in the treatment of human diseases [1,10,11]. The most well-known inhibitors of PTPs are inorganic compounds, for example sodium orthovanadate [12,13], nitric oxide [14], or phenylarsine oxide [15]. The major disadvantages of these compounds are their lack of specificity and serious side effects possession. The vast majority of the described PTPs inhibitors does not have drug-like properties with low permeability, selectivity, and cell efficiency. Numerous studies have been conducted with the aim of designing new, more potent and selective PTPs inhibitors. The main problem of all these investigations is the high sequence homology of the catalytic center shared by all protein tyrosine phosphatases [16]. The identification of a new binding site for PTPs, which is less conservative among phosphatases, constitutes a new paradigm for inhibitor design. Many researchers have indicated, that potential inhibitory compounds can bind both to the catalytic center and a secondary binding site, inducing allosteric inhibition [17].

Vanadium compounds have been shown to improve insulin levels and reduce the symptoms of diabetes. In the first phase of clinical studies, two inorganic salts, vanadyl sulfate and sodium metavanadate, were evaluated. Type 2 diabetes and obesity are both characterized by insulin and leptin resistance, which are hormones produced by the pancreas. According to current theories, the enzyme PTP1B functions as a negative regulator of insulin and leptin signal transduction and could be a pharmacological target for the treatment of these disorders [18,19]. Unfortunately, due to poor absorption and severe gastrointestinal irritation, they found no anti-diabetic application. However, modification of these complexes by the introduction of organic ligands may lead us to a new therapeutic strategy. Specific ligands can modulate vanadium complexes by increasing the potency of inhibition of the phosphatase enzyme. As a result, a design of selective inhibitor of PTP1B with therapeutic properties is potentially possible [20,21].

The anticancer activity of four oxovanadium(IV) complexes and one dioxovanadium(V) complex against the MCF-7 breast cancer cell line, the MDA-MB-231 triple-negative breast cancer cell line, and the human keratinocyte HaCaT cell line is presented in this paper. All of the studied compounds were shown to possess the inhibitory properties against PTP1B, which may lead to their anticancer potential. We performed a computer simulation of their binding inside the active region of PTP1B to obtain a better understanding of the relationship between the structure of the studies compounds and their activity. What is important, in this study we showed a correlation between PTP1B inhibitory properties and anticancer efficacy against breast cancer cell lines. This comparison may be useful in the development of novel vanadium complexes-based medicines, since the majority of this type of research has, in recent years, focused more on the treatment of diabetes, obesity, and insulin resistance.

## 2. Results

### 2.1. Synthesis

**Figure 1:**
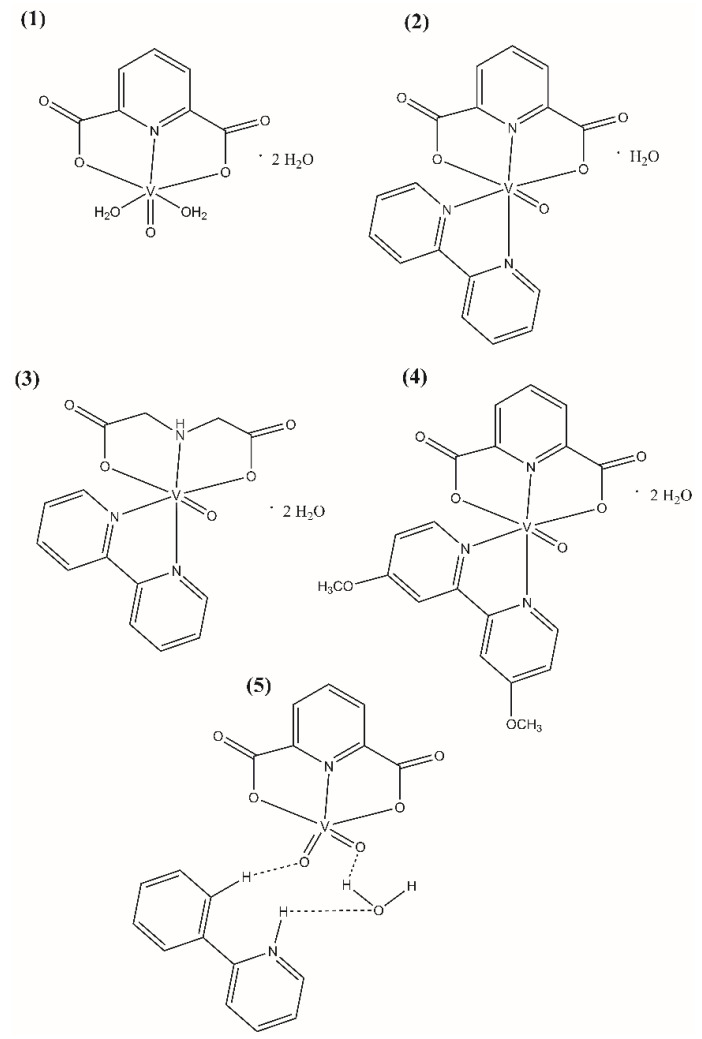
Molecular formulas of (**1**) [VO(dipic)(H_2_O)_2_]·2 H_2_O, (**2**) [VO(dipic)(bipy)]·H_2_O, (**3**) [VO(ida)(bipy)]·2 H_2_O, (**4**) [VO(dipic)(dmbipy)]·2 H_2_O, (**5**) [VOO(dipic)](2-phepyH)·H_2_O.

The polycarboxylate oxovanadium(IV) and dioxovanadium(V) complex compounds (Figure 1) were synthesized according to the procedures described in the literature: [22,23] for [VO(dipic)(H_2_O)_2_]·2 H_2_O (Compound **1**), [VO(dipic)(bipy)]·H_2_O (Compound **2**) and [VOO(dipic)](2-phepyH)·H_2_O (Compound **5**), [24] for [VO(ida)(bipy)]·2 H_2_O (Compound **3**), [25] [VO(dipic)(dmbipy)]·2 H_2_O (Compound **4**). The complex compound [VOO(dipic)](2-phepyH)·H_2_O was synthesized in crystalline form and all data specifying the XRD analysis of this complex have been described in the publication [22].

### 2.2. Inhibitory Activity against PTP1B Phosphatase

We investigated the inhibitory potential of Compounds **1**–**5** and sodium orthovanadate (in the concentration range of 39.0625 nM to 5 µM) and additionally 2,6-pyridinedicarboxylic acid (dipic), 2,2′-bipyridine (bipy), iminodiacetic acid (ida), 4,4′-dimethoxy-2,2′-bipyridine (dmbipy) and 2-phenylpyridine (2-phephy) (in concentration range of 500 nM to 5 µM) against protein tyrosine phosphatase PTP1B. 

**Figure 2:**
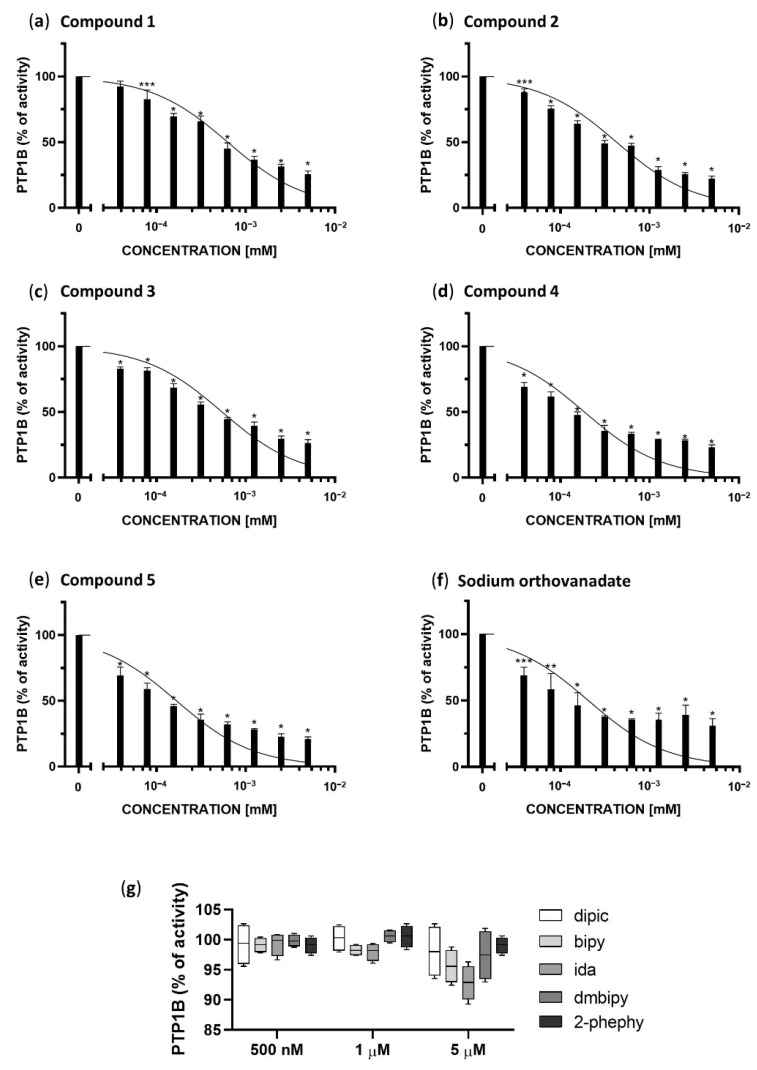
Enzymatic activity of PTP1B after treatment with different concentrations of Compounds **1**–**5** (**a**–**e**), sodium orthovanadate, as a standard inhibitor of PTP1B (**f**), and ligands present in the coordination sphere of the studied vanadium(IV,V) complexes (dipic, bipy, ida, dmbipy, 2-phephy) (**g**). The results are presented as a percentage of the control as means ± SD (*n* = 3, * *p* < 0.0001; ** *p* < 0.001; *** *p* < 0.01).

As shown in Figure 2, each of the examined complexes (a–e) and sodium orthovanadate (f) but not the ligands themselves (g), inhibited the enzymatic activity of PTP1B phosphatase in a concentration-dependent manner. We calculated the IC_50_ for each complex, and compared them to the inhibitory properties of sodium orthovanadate, which is known as a standard inhibitor of PTP1B. Moreover, based on the results of molecular docking we have defined the inhibitory type as competitive, therefore we calculated Ki values for the inhibition of PTP1B based on following equation:

Ki = IC_50_/(S/Km + 1)

where:

IC_50_–The obtained half maximal inhibitory concentration for each complexes;
[S]–substrate concentration (1 mM);
[Km] = The calculated Michaelis constant (0.7 mM).

The obtained results are summarized in Table 1.

All compounds in the concentration range of 1.25–5 µM show a similar inhibitory effect between 80–60%. Compounds **4** and **5**, the visible changes in the inhibition of PTP1B in relation to the other compounds occur in the concentration range of 39,063 to 625 nM. These compounds show the best inhibitory properties, inhibiting the enzyme in 50% at a concentration of 185.4 ± 9.8 and 167.2 ± 8.0 nM, respectively, and their half maximal inhibitory concentration is lower than for standard inhibitor of PTP1B phosphatase, sodium orthovanadate (204.1 ± 25.15 nM). Sodium orthovanadate and Compound **4** in the concentration range 39.063 to 625 nM show similarly inhibition in the range 31–67%, whereas Compound **5** shows 38–68%. The remaining compounds are also effective at the low concentrations tested. However, when comparing to the results for the lowest statistically significant concentrations tested (78.125 nM), Compounds **1**–**3** show an inhibitory effect in 17% (*** *p* <0.01), 19% (* *p* <0.0001) and 25% (* *p* <0.0001) respectively.

### 2.3. In Silico Studies

**Figure 3:**
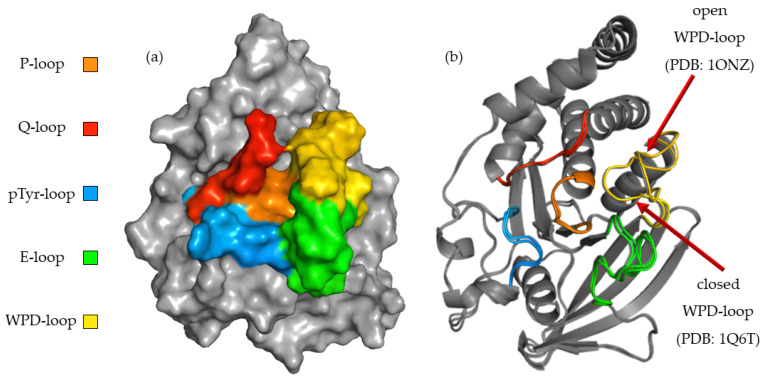
(**a**) Surface representation of PTP1B (PDB: 1ONZ) with color-coded active site loops. (**b**) Comparison of WPD-loop conformation observed in 1Q6T complex (closed position) and 1ONZ (open position).

To better understand the relationship between the structure of the compounds tested and their activity, we performed a computer simulation of binding within the active site of PTP1B (Figure 3). P-loop, which is also referred as PTP-loop, is the central part of the active site. It contains Cys215 and Arg221 residues, which are crucial for the dephosphorylation processes. P-loop is a major binding site for orthovanadate and many other inhibitors [26,27]. P-loop is surrounded by four loop structures, such as WPD, Q-loop, pTyr-loop and E-loop responsible for ligand binding, substrate recognition and taking part in catalysis functions [28].

The high flexibility of the WPD-loop plays an important role in the rate-limiting step for hydrolysis and radically changes the shape of the PTP1B active site. Asp181 after closure of the WPD-loop participates in two crucial steps of dephosphorylation: the creation of phosphoenzyme and the hydrolysis of cysteinyl-phosphate bonds [29]. Some inhibitors, like orthovanadate, stabilize it in a closed (hydrolysis competent) position [30]. Other compounds promote open (hydrolysis incompetent) conformation of the WPD-loop [29]. To learn about the binding preferences of the tested compounds, we used complexes with the WPD-loop in the closed and open positions during docking experiments. The final results showed a clear binding preference to crystals in which the WPD-loop was in the open position. This was most noticeable with ligands containing 2,2′-bipyridine in their structure. During binding to the 1ONZ and 3EAX complexes, the GoldScore values for individual ligands were higher than those obtained during docking to the complexes with the loop in the closed position. We applied additional rescoring in which we predicted the change of free energy (ΔG) and predicted Ki for each of the obtained final poses. Although the GS function values were not strongly correlated with the values of the predicted ΔG (R2 = 0.61), they also indicated a stronger interaction of the studied ligands with the WPD open-loop complexes. KDEEP predicted a lower binding strength of the tested ligands to PTP1B than that obtained in biological studies, however similar to the GS score, it correctly ordered the activity of the compounds based on the docking results to the 1ONZ complex. Moreover, the mean RMSD values for the three highest rated poses show a much greater reproducibility of the docking results for complexes with WPD-loop in open conformation. The exact values of the scoring function and mean RMSD are summarized in Table 2. In the case of docking to the other complexes, the highest rated poses were above the Q and pTyr loops, limiting access to the active site only to a small extent and the results were very difficult to recreate between each docking run.

Because of the high convergence of the scoring function values with the results of in vitro studies and the very high reproducibility of docking poses, we chose 1ONZ complex as a model structure during the description of binding modes of studied ligands.

**Figure 4:**
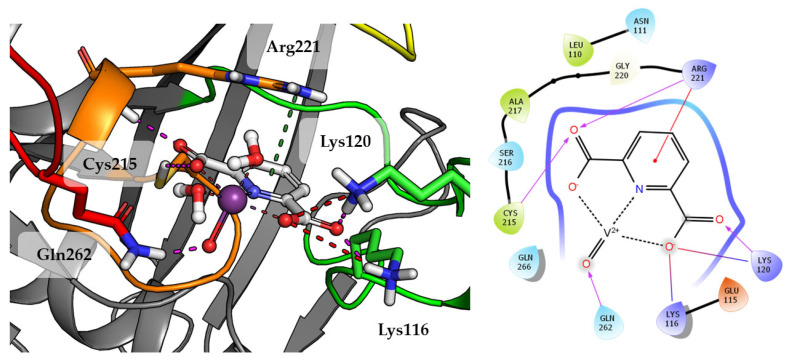
Docking result of Compound **1** to the PTP1B active site (PDB: 1ONZ). Left panel presents a studied compound as white sticks with protein represented by a gray cartoon. Significant loops are highlighted with separate colors; P-loop in orange, WPD-loop in yellow, E-loop in green, and Q-loop in red. The most important amino acids are represented as sticks with the same color code. Coordinate bonds are marked with thin dotted lines. Hydrogen bonds are marked in magenta, ionic interaction in red, aromatic π-π or CH-π in green. Right panel presents 2D ligand interaction diagram.

Compound **1** which is the smallest among the tested compounds, creates strong interactions within P-loop (Figure 4). Both amino acids crucial for dephosphorylation processes, Cys215 and Arg221 create hydrogen bonds with one first acidic group of pyridine-2,6-dicarboxylic acid. Arg221 take part in cation-π interaction with pyridine. Second carboxylic group creates salt bridges and hydrogen bonds with Lys116 and Lys120 from E-loop. The molecule is additionally stabilized by the hydrogen bond between vanadium oxide and Gln262.

**Figure 5:**
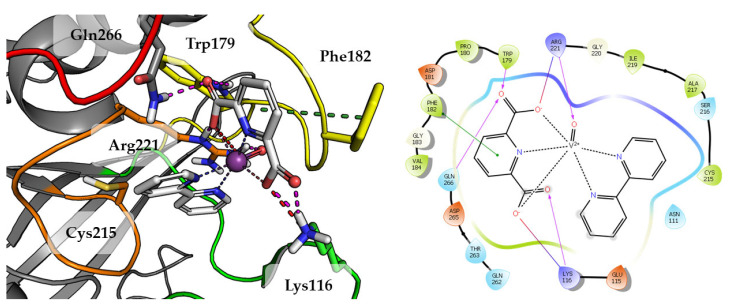
Docking result of Compound **2** to the PTP1B active site (PDB: 1ONZ). Left panel presents a studied compound as white sticks with protein represented by a gray cartoon. Significant loops are highlighted with separate colors; P-loop in orange, WPD-loop in yellow, E-loop in green, and Q-loop in red. The most important amino acids are represented as sticks with the same color code. Coordinate bonds are marked with thin dotted lines. Hydrogen bonds are marked in magenta, ionic interaction in red, aromatic π-π or CH-π in green. Right panel presents 2D ligand interaction diagram.

Substitution of aqua Compound **1** by 2,2′-bipyridine in Compound **2** leads to an increase in the number of observed interactions. This was also observed in a higher GoldScore value 51.52. As shown in Figure 5, DIPIC binds strongly with the E-loop and WPD-loop. One of the carboxyl groups creates many polar interactions with Arg221, Trp179 and Gln266. Other carboxyl group created a salt bridge with Lys116. The aromatic interaction of the piperidine ring with Phe182 stabilizes the position of compound. Even oxygen from oxovanadium(IV) cation is involved in the hydrogen bond with Arg221.

**Figure 6:**
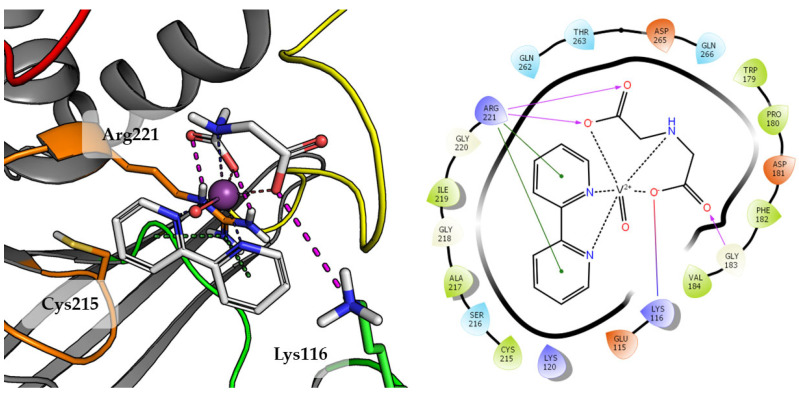
Docking result of Compound **3** to the PTP1B active site (PDB: 1ONZ). Left panel presents a studied compound as white sticks with protein represented by a gray cartoon. Significant loops are highlighted with separate colors; P-loop in orange, WPD-loop in yellow, E-loop in green, and Q-loop in red. The most important amino acids are represented as sticks with the same color code. Coordinate bonds are marked with thin dotted lines. Hydrogen bonds are marked in magenta, ionic interaction in red, aromatic π-π, CH-π and cation-π in green. Right panel presents 2D ligand interaction diagram.

Compound **3** was one of the lower rated by scoring function (GoldScore value 48.04), what correlates with the results of biological studies. The final pose of Compound **3** is shown in Figure 6.

Compound **3**, the 2,2′-bipyridine fragment may approach Arg221 more easily by creating cation-π interactions. The iminodiacetic acid fragment forms a salt bridge with Arg221 and ionic interactions with Lys116. Oxovanadium(IV) cation appears to be not involved in any specific interactions with active site amino acids.

**Figure 7:**
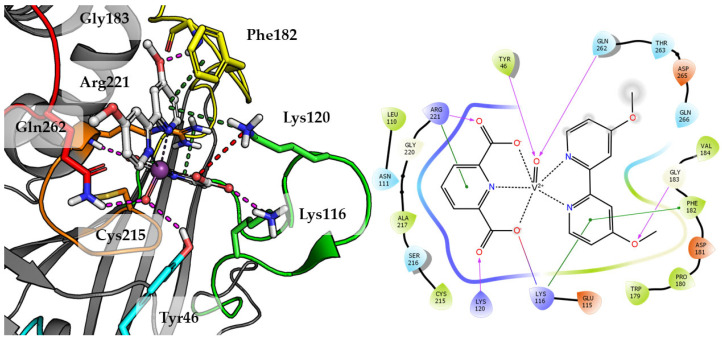
Docking result of Compound **4** to the PTP1B active site (PDB: 1ONZ). Left panel presents a studied compound as white sticks with protein represented by a gray cartoon. Significant loops are highlighted with separate colors; P-loop in orange, WPD-loop in yellow, E-loop in green, and Q-loop in red. The most important amino acids are represented as sticks with the same color code. Coordinate bonds are marked with thin dotted lines. Hydrogen bonds are marked in magenta, ionic interaction in red, aromatic π-π, CH-π and cation-π in green. Right panel presents 2D ligand interaction diagram.

Top scored docking results of Compound **2** (Figure 7) presented little contribution of the 2,2′-bipyridine fragment to binding in PTP1B active site. Introducing methoxy groups into 2,2′-bipyridine changed the orientation of Compound **4**, significantly improving the alignment of this fragment between obtained poses and overall binding with the increase of GoldScore value to 60.94, the highest among all docked compounds.

The methoxy group leads compound closer to the WPD loop by participating in hydrogen bond with the protein backbone (Gly183). As a result, one of the pyridine ring from 2,2′-bipyridine system could take part in the aromatic interaction of π-π with Phe182 and cation-π with Lys116. At the same time, DIPIC was in a very convenient position, on the one hand, creating hydrogen bonds and cation-π with P-loop Arg221 and, on the other, a series of polar interactions with Lys116 and Lys120 from E-loop. This arrangement of the compound significantly improved interactions with vanadyl, the oxygen atom of which is involved in hydrogen bonds with Tyr46 and Gln262.

**Figure 8:**
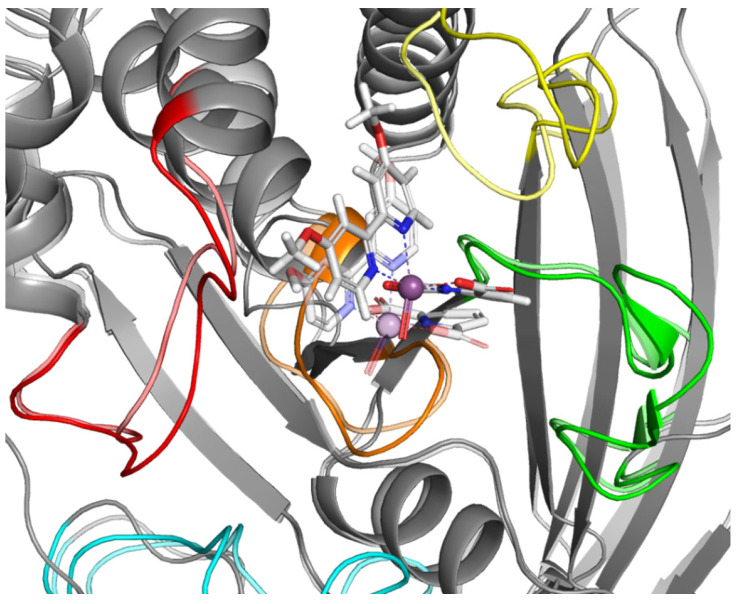
Result of MD simulation of Compound **4** (white sticks) and the PTP1B complex (1ONZ). Transparent structures presents a studied complex on start of simulation and filled structures present position after 50 ns. Significant loops are highlighted with separate colors; P-loop in orange, WPD-loop in yellow, E-loop in green, and Q-loop in red.

Docking results showing the preference of the tested ligands to interact with complexes in which the WPD loop was in the open position. On this basis, we assumed that the stabilization of the open conformation might be how the test compounds exert their effects. To verify this hypothesis, we conducted further studies involving molecular dynamics (MD) simulations of Compound **4**. The obtained results are in line with our assumptions (Figure 8). 

**Figure 9:**
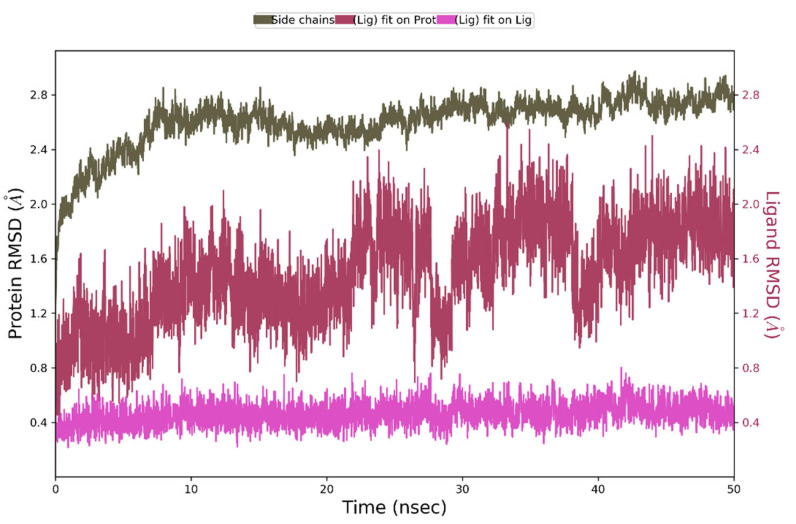
The above plot shows the RMSD evolution of a protein backbone (green) and Compound **4** (aligned to its reference conformation – pink, aligned on the protein backbone of the reference position - magenta) calculated for all frames in the simulation.

During 50 ns of MD simulation, the changes observed in the studied complex were minimal, with RMSD value less than 1Å for ligand alignment to its reference conformation and only 2.7 Å deviation from its starting position (Figure 9).

**Figure 10:**
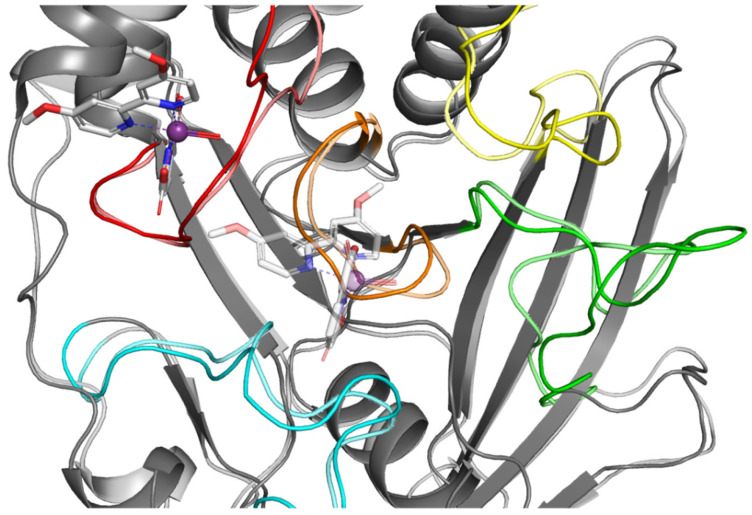
Result of MD simulation of Compound **4** (white sticks) and the PTP1B complex (2FJN). Transparent structures present a studied complex on start of simulation and filled structures present position after 50 ns. We highlight significant loops with separate colors; P-loop in orange, WPD-loop in yellow, E-loop in green, and Q-loop in red.

For comparison, we performed analogous simulations in which the starting point was the highest rated ligand **4** poses from the WPD closed-loop PTP1B protein (2FJN). In both cases, the ligand has diffused away from its initial binding site during the course of the simulation (Figure 10). 

**Figure 11:**
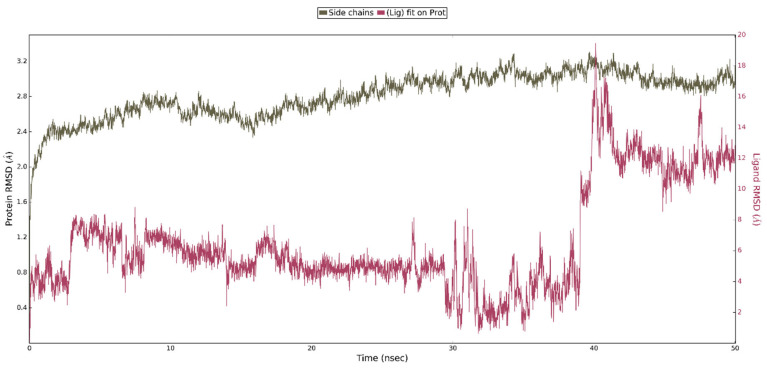
The above plot shows the RMSD evolution of a protein backbone (green) and ligand **4** aligned with the protein backbone of the reference position - magenta calculated for all frames in the simulation.

This shows that interaction of ligand **4** with the WPD closed-loop complex is much weaker and cannot be responsible for enzyme inhibition. Analysis of the RMSD plots for the performed simulation shows the gradual movement of the ligand away from the initial binding site. (Figure 11). The main protein chain in this complex undergoes more changes than in the previously analyzed one. However, we were unable to observe the opening of the WPD loop because of interaction with Compound **4** during the 50 ns simulation.

**Figure 12:**
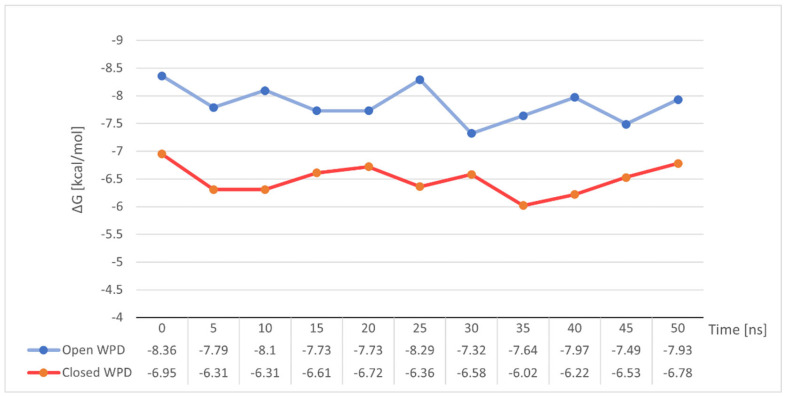
Changes in binding free energy for Compound **4** during MD simulation measured at 5 ns intervals.

To our surprise, the variation of free energy in both simulations did not show any significant changes (Figure 12). Both in the case of stable binding of Compound **4** to open-loop WPD PTP1B and in the complex with closed WPD in which we observed a clear drift of the compound beyond the active site of the protein, mean ΔG values were very close to those determined for the post-docking positions. −7.85 kcal/mol in the 1ONZ complex and −6.49 kcal/mol in 2FJN complex. In the first case, changes in ΔG seem to correlate with the oscillation of the WPD open loop, which during the interaction slightly moves away from the ligand to come back again, strengthening the ligand-protein interaction. In the case of Compound **4** docked to the PTP1B protein in which the WPD loop was closed, the decrease in ΔG value to −6.02 kcal/mol coincides with the moment of instability seen in major RMSD changes from its initial position (Figure 11 between 30 and 40 ns). This is when the Compound **4** moves away from the active center of PTP1B. The subsequent increase in free binding energy was related with the creation of new interactions with Tyr20, Arg24 and Arg254 (the final position of the compound shown on Figure 10)

### 2.4. Cytotoxicity against Breast Cancer Cell Lines and Human Keratinocyte Cell Line

We performed the cytotoxicity studies of the synthesized Compounds **1**–**5** and sodium orthovanadate (as a standard inhibitor of PTP1B) against the MCF-7 and MDA-MB-231 breast cancer cell lines, as well as the human keratinocyte HaCaT cell line (as a noncancerous control). Cells were treated with serial concentrations of each substance (390.0625 nM to 100 µM) and then incubated for 24 h. We observed that all the synthesized complexes tested are effective in inhibiting both the enzymatic activity of PTP1B phosphatase and the viability of the evaluated cell lines. On the other hand, sodium orthovanadate, which is a well-known inhibitor of phosphatases, shows significantly weaker cytotoxic effect than synthesized compounds.

**Figure 13:**
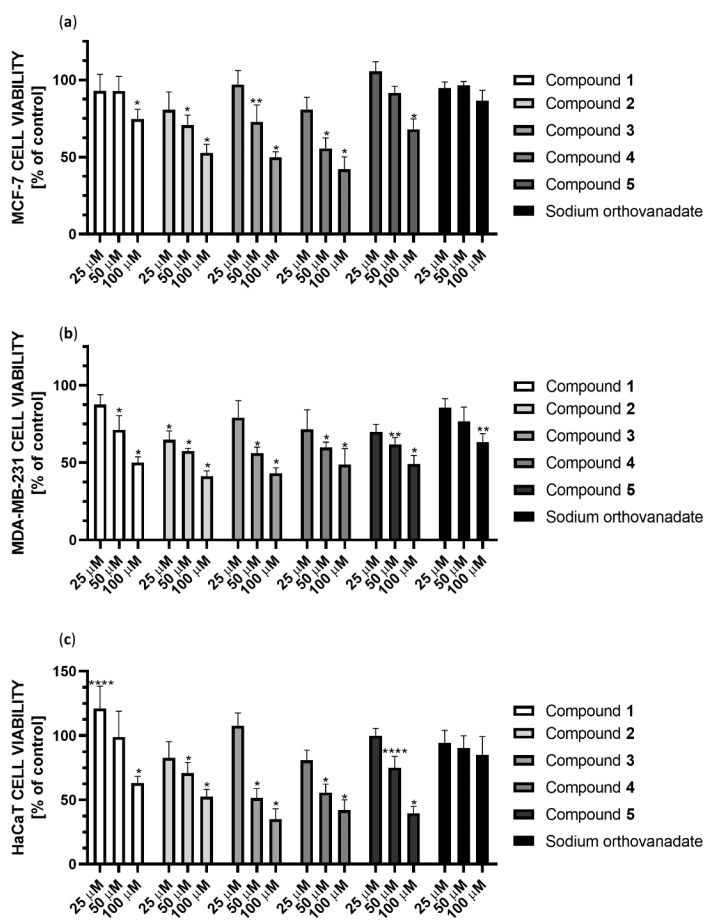
Cytotoxicity of Compounds **1**–**5** and sodium orthovanadate at concentrations 25, 50, and 100 µM against (**a**) the breast cancer MCF-7 cell line; (**b**) the breast cancer MDA-MB-231 cell line; (**c**) the human keratinocyte HaCaT cell line. Cell viability was measured by the MTT cell viability assay. The results were presented as a percentage of the control (mean ± SD, *n* = 3, * *p* < 0.0001; ** *p* < 0.001; **** *p* < 0.1).

The cytotoxic effect of the studied complexes was observed at concentrations ranging from 25 to 100 µM (Figure 13). Compound **4** showed the strongest cytotoxic effect against MCF-7 cells, reducing cell viability in this concentration range to 86%, 61% and 53%, respectively. In the case of MDA-MB-231 cells, Compound **2** showed the strongest cytotoxic effect in this concentration range, reducing cell viability to 65%, 56%, and 44%, respectively. Subsequently, Compound **3** showed a similar effect, decreasing cell viability to 76%, 55%, and 46%, respectively. Both complexes contain the 2,2′-bipyridine ligand in their structure. Comparing the cytotoxicity results for Compounds **4** and **5** at 25 µM, they reduced cell viability to 75% and 71%, respectively, which may correlate with the inhibition results against PTP1B. Unfortunately, all compounds also show cytotoxicity to non-tumor transformed cells HaCaT cell line. However, Compound **1** is noteworthy because it improved cell growth at low concentrations. At 50 µM concentration, it showed no cytotoxic effect in HaCat cells, while it showed a 25% cytotoxic effect in MDA-MB-231 cells.

## 3. Discussion

Recent therapeutic methods in the field of generating new cancer treatments have focused on regulating gene transcription products, particularly the regulation of cancer-specific signaling pathways. Some of these pathways may be completely unique for cancer cells, with no effect on cells in a normal state. In recent years, the creation of coordination molecules with desirable features has attracted the interest of researchers who have become more focused on this area. Complex compounds that possess several functionally significant features are of great significance. The properties of the central atom and the ligands should be taken into consideration when evaluating such complex compounds. Chemical compounds containing heterocyclic organic ligands have been identified as being among one of the most promising ligands for the formation of multifunctional complexes. To gain insight into the relationship between the structure of the complexes and their physicochemical, biological, and biomedical properties, it is necessary to test the synthesized compounds using the broadest possible spectrum of research tools. As a result of this research, not only chemists, but also biochemists and biomedicists can benefit from the results.

The discovery that metals are required for the structure and function of biomolecules, completely changes the viewpoint of scientists on the role of metal ions in living organisms. The literature study demonstrated that the chemical form (oxidation state of vanadium, ligand type, and geometric arrangement) of vanadium-based complexes determines their physicochemical and consequently biological features. The chemical nature of vanadium-based complexes can affect the affinity for DNA, oxidative stress, or the type of cell death induced in cancer cells [31]. Because vanadate has a structural similarity to phosphate, it has been proposed as a phosphate transition state analog in phosphatases. Therefore, vanadium compounds are of great interest as antidiabetic agents and, more recently, as potential anticancer drugs [32,33,34].

The dipicolinate and iminodiacetate complexes oxovanadium(IV) and dioxovanadium(V) are known for their antioxidant properties towards the superoxide radical anion. The presence of aromatic ligands in the coordination spheres of oxovanadium(IV) and oxovanadium(V) increases the antioxidant properties. Complexes **1** [VO(dipic)(H_2_O)_2_]·2 H_2_O, **2** [VO(dipic)(bipy)]·H_2_O and **3** [VO(ida)(bipy)]·2 H_2_O have equivalents of l-ascorbic acid 2.359, 0.936 and 5.43, respectively [22,24]. Among the dipicolinate complex compounds, Compound **5** [VOO(dipic)](2-phepyH)·H_2_O deserves attention because it has better antioxidant activity towards superoxide anion radicals than l-ascorbic acid. The calculated equivalent towards l-ascorbic acid for [VOO(dipic)](2-phepyH)·H_2_O is 0.68 [22]. On the other hand, antioxidants might act as pro-oxidants when utilized in excessive amounts. The generation of hydrogen peroxide, hydroxyl radicals, and other intermediates of this process constitutes the pro-oxidative effect [35]. The role of oxidative stress in the cytotoxic activity of vanadium-based compounds has been well established in the literature. Complexes containing vanadium(IV) in the coordination center increased ROS levels [31]. In our research presented in this article, Compounds **1**–**4** contain vanadium(IV), whereas Compound **5** contains vanadium (V). As a result, there may be a difference in cytotoxicity against MCF-7 breast cancer cells.

The estrogen receptor (ER), the progesterone receptor (PR) and the human epidermal growth factor 2 receptor (HER2) are absent in triple negative breast cancer (TNBC), making it one of the most aggressive types of breast cancer (HER2). As a result, there are no targeted drugs available for the treatment of TNBC at this time-standard chemotherapy is the primary systemic treatment. Patients with TNBC demonstrate greater chemosensitivity compared to patients with other types of breast cancer. TNBC tumors, on the other hand, have a strong potential to metastasize, resulting in a worse prognosis for recovery [36]. PTP1B stimulates the growth of HER2 positive and triple negative cell lines while inhibiting apoptosis in the same cells. As a result, inhibition of PTB1B has the ability to promote apoptosis of cancer cells in vitro [8,11,37]. For this reason, based on our research, it seems possible that inhibition of PTP1B in MDA-MB-231 cells causes a stronger cytotoxic effect than in MCF-7 cells.

Biochemical and structural studies have provided a complete understanding of the catalytic process for the PTP catalytic domain. PTP signature motif, WPD-loop with conserved and catalytically important aspartate residue, which is important due to its role in enzyme dephosphorylation, and phosphotyrosine recognition loop are only a few notable features of this domain that need special mention [17]. In our study results obtained from molecular docking may suggest that the tested compounds inhibit the action of PTP1B mainly due to the stabilization of the WPD-loop in the open conformation, unlike the sodium orthovanadate which binds mostly within the P-loop [26,27].

## 4. Materials and Methods

### 4.1. Chemicals and Reagents

2,6-pyridinedicarboxylic acid (dipic), 4,4′-Dimethoxy-2,2′-bipyridine (dmbipy), Dimethylsulfoxide (DMSO), Fetal bovine serum (FBS), 3-(4-dimethylthiazol-2-yl)-2,5-diphenyltetrazolium bromide (MTT), *para*-nitrophenyl phosphate (pNPP) was purchased from Sigma-Aldrich. 2,2′-Bipyridine (bipy) and 2-Phenylpyridine (2-phephyH) was purchased from Merck. Iminodiacetic acid (ida) was purchased from Acros Organics, Geel, Belgium. Sodium orthovanadate was purchased from Ambeed, Arlington Heights, IL, USA. Dulbecco’s Modified Eagle’s Medium (DMEM) and Phosphate-buffered saline (PBS) were purchased from PAN-biotech, Aidenbach, Germany. Recombinant PTP1B phosphatase was purchased from Cayman Chemical, Ann Arbor, MI, USA.

### 4.2. Synthesis

The elemental analysis for synthesized complex compounds of oxovanadium(IV) and dioxovanadium(V) are as follows: for [VO(dipic)(H_2_O)_2_]·2 H_2_O: 27.64% C, 3.60% H, and 4.70% N; and analysis calculations: 27.63% C, 2.96% H, and 4.61% N; for [VO(dipic)(bipy)]·H_2_O: showed 49.99% C, 3.35% H, and 10.29% N; analysis calculations 50.25% C, 3.20%: H, 10.34% N; for [VO(ida)bipy]·2 H_2_O: 43.08% C, 4.36% H, 10.77% N, and analysis calculations: 43.08% C, 4.36% H, 10.74% N; for [VO(dipic)(dmbipy)]·2 H_2_O 47.46% C, 3.69% H, and 8.77% N; and analysis calculations 47.10% C, 3.93% H, and 8.68% N.

The complex compound [VOO(dipic)](2-phepyH)·H_2_O was synthesized in a crystalline form and all data specifying the XRD analysis of this complex have been described in the publication [22].

### 4.3. Recombinant PTP1B Assay

The experiment was carried out on 96-well plates. The final concentration of PTP1B phosphatase in the reaction samples was 0.8 g/mL (10 nM). The enzyme was treated with solvent (as a control), Compounds **1**–**5** or sodium orthovanadate (as a standard inhibitor of PTP1B) at concentrations ranging from 39.0625 nM to 5 µM or 2,6-pyridinedicarboxylic acid (dipic), 2,2′-bipyridine (bipy), iminodiacetic acid (ida), 4,4′-dimethoxy-2,2′-bipyridine (dmbipy) and 2-phenylpyridine (2-phephy) in the concentration range 500 nM to 5 µM. The enzyme activities of PTP1B phosphatase were evaluated at 37 °C using 1 mM chromogenic substrate *para*-nitrophenyl phosphate (*p*NPP) in 10 mM HEPES buffer pH 7.4. The increase in absorbance (due to the formation of *para*-nitrophenols) is linearly proportional to the concentration of enzyme activity (with excessive substrate, i.e., zero-order kinetics) and was measured at 405 nm using DigiRead Communication Software (version 1.2.0.2., Dan Kittrich, Asys Hitech GmbH, Eugendorf, Austria) on the Jupiter microplate reader (Biogenet, Józefów, Poland). Data were expressed as the percentage of untreated enzyme (control).

### 4.4. Cell Lines and Culture

The MCF-7 and MDA-MB-231 breast cancer cell lines and the human keratinocyte HaCaT cell line were obtained from the American Type Culture Collection (ATCC, Manassas, VA, USA). Dulbecco’s modified eagle medium supplemented with 10% fetal bovine serum (FBS) and 1% penicillin/streptomycin was used to cultivate the cell lines. The cells were cultured at 37 °C in a 5% CO_2_ atmosphere.

### 4.5. Cell Viability Assay

According to the general protocol, a cell viability assay (MTT assay) was performed. Briefly, cells (1.2 × 10^6^ cells/mL) were treated with solvent (control) or serial solutions of Compounds **1**–**5** or sodium orthovanadate at concentrations ranging from 390.0625 nM to 100 µM. The chemicals were dissolved in a growth medium containing 1% penicillin/streptomycin. After 24 h, 0.5 mg/mL of MTT solution (3-[4,5-dimethylthiazol-2-yl]-2,5-diphenyl-tetrazolium bromide) was added. 100 µL of DMSO was added to each well once the purple precipitate was clearly visible under the microscope. A microplate reader (Biogenet, Jozefow, Poland) and DigiRead Communication Software were used to measure the absorbance at 492 nm (Asys Hitech GmbH, Eugendorf, Austria). Data were expressed as the percentage of untreated cells (control).

### 4.6. Molecular Modeling

We chose Compounds **1** to **4** for in silico experiments. Because of the complex structure of Compound **5** and the interaction of dioxo-(pyridine-2,6-dicarboxylato)-vanadium(V) fragment with 2-phenylpyridine through hydrogen bonds (including via a water molecule) it was impossible to determine the molecules involved in binding to the protein.

The three-dimensional structures of compounds were prepared in the Maestro 11.2, (Schrödinger) program. The Sybyl-X 2.1.1, (Tripos) was used to assigning partial charges and determine the correct atom types.

Due to the high flexibility of the PTP1B active site, the analysis used five complexes available in the PDB (Protein Data Base) with the codes 1ONZ, 1Q6T, 2FJN, 3EAX and 5KAD.

All proteins were prepared using the ProteinPreparation Wizard tool (Maestro-Schrödinger), thanks to which appropriate charges and conformation of amino acid residues were assigned. Additionally, the hydrogen bond network was adjusted for pH 7.4.

Prepared complexes were used for molecular docking. All amino acids within 15 Å of the Cys215 sulfur atom were taken as the binding site. The GOLD 5.3 (CCDC) program was used for docking studies using the predefined GA settings, GoldScore evaluation function during docking and ChemScore during rescoring. Docking of each ligand was performed ten times, keeping all poses obtained. Mean RMSD was calculated for the three highest-rated poses.

After docking each ligand to all proteins, the highest scored pose was optimized to better reflect the interactions with PTP1B using the Refine Protein-Ligand Complex tool (Maestro-Schrödinger).

The final complexes of ligands docked to the PTP1B protein were re-evaluated with the KDEEP [38] web service, which predicted their Ki values.

For the most active of the modeled ligands, compound number **4**, we performed 50ns molecular dynamics simulation to compare the stability of the complex formed after docking to the protein from the WPD loop in the open conformation (1ONZ) and in the closed conformation (2FJN). Both the preparation of the complexes and the simulation itself were carried out using the Desmond 5.0 program implemented in the Maestro Schrödinger package [39]. The orthorhombic box-shaped system was filled with water molecules (TIP3P solvent model), 0.15M NaCl and neutralized with an appropriate amount of additional Na^+^ ions. The OPLS3 force field was used for its preparation. Before starting the actual simulation, the complex was minimized (100ps). Before starting the actual simulation, the complex was minimized (100ps). The simulation itself was run for 50ns using NPT ensemble class and prior system relaxation. 5000 frames were collected during the simulation (10ps recording interval).

The results were visualized with the PyMol 2.4.1 program (Schrödinger) and Ligand interaction diagram (Schrödinger).

## 5. Conclusions

The development of research on protein tyrosine phosphatase inhibitors may contribute in the discovery of novel therapeutic strategies. In the opinion of both the World Health Organization and the Union for International Cancer Control, cancer will soon overtake all other causes of death to take the first place on the list. The sodium orthovanadate is an inorganic compound and the best known protein tyrosine phosphatase inhibitor. However, inorganic PTPs inhibitors are mainly not specific; they may have an impact on a broad spectrum of human enzymes, which is their primary limitation. In this study, we present five vanadium compounds with anticancer efficacy against the MCF-7 breast cancer cell line, the MDA-MB-231 triple-negative breast cancer cell line, and the human keratinocyte HaCaT cell line. We discovered that all examined compounds are PTP1B inhibitors, what corresponds with their anticancer activity. To further understand the relationship between compound structure and activity, we computer simulated their binding inside PTP1B’s active region. In contrast to sodium orthovanadate, which binds entirely within the P-loop, the results of our investigation show that the tested chemicals limit the function of PTP1B mostly due to the stability of the WPD-loop in the open conformation. The obtained results can be used in a future to increase the effect of anticancer systemic drugs and improving its inhibitory effects and antiproliferative properties.

## Figures and Tables

**Table 1:** IC_50_ and Ki values against PTP1B for Compounds **1**–**5** and sodium orthovanadate.

Compounds	IC_50_ ± SD [nM]	Ki ± SD [nM]
**1**	627.5 ± 39.1	256.3 ± 15.3
**2**	426.3 ± 37.1	173.4 ± 17.2
**3**	542.8 ± 48.4	221.4 ± 19.1
**4**	185.4 ± 9.8	74.2 ± 4.1
**5**	167.2 ± 8.0	66.7 ± 3.3
Sodium orthovanadate	204.1 ± 25.15	96.8 ± 15.6

**Table 2:** GoldScore (GS) values, predicted ΔG [kcal/mol], Ki [µM] and mean RMSD (gray column) for Compounds **1**–**4** docked to complexes with WPD-loop in open and closed position.

**Open**												
**1ONZ**							**3EAX**					
	**GS**	**ΔG** [kcal/mol] *			Ki[µM] *	**RMSD**	**GS**	ΔG[kcal/mol] *		Ki[µM] *	**RMSD**	
**1**	40.80	−5.03			147.91	0.1	38.86	−5.81		48.98	0.16	
**2**	51.52	−7.65			1.23	0.12	48.42	−7.08		4.57	5.19	
**3**	48.04	−6.68			7.94	0.62	46.65	−6.57		9.55	5.48	
**4**	60.94	−8.36			0.28	0.09	45.56	−7.74		1.17	6.93	
**Closed**												
	**1Q6J**				**2FJN**				**5KAD**			
	**GS**	ΔG[kcal/mol] *	Ki[µM] *	**RMSD**	**GS**	ΔG[kcal/mol] *	Ki[µM] *	**RMSD**	**GS**	ΔG[kcal/mol] *	Ki[µM] *	**RMSD**
**1**	34.84	−4.66	263.03	0.21	33.94	−5.08	173.78	13.29	32.60	−5.77	54.95	9.35
**2**	18.17	−5.17	151.36	16.64	30.26	−5.95	18.62	13.08	37.61	−6.49	8.51	4.24
**3**	24.38	−5.42	56.23	14.98	32.05	−5.55	60.26	17.17	32.11	−5.49	53.70	6.98
**4**	37.55	−7.28	1.95	5.01	41.39	−6.95	4.37	7.66	43.66	−7.08	2.57	6.3

* Values predicted with KDEEP web service.

## Data Availability

Data is contained within the article.
